# A new measure for the strength of electrical synapses

**DOI:** 10.3389/fncel.2015.00378

**Published:** 2015-09-25

**Authors:** Julie S. Haas

**Affiliations:** Department of Biological Sciences, Lehigh UniversityBethlehem, PA, USA

**Keywords:** gap junction, electrical synapses, efficacy, thalamic reticular nucleus, connexin36

## Abstract

Electrical synapses, like chemical synapses, mediate intraneuronal communication. Electrical synapses are typically quantified by subthreshold measurements of coupling, which fall short in describing their impact on spiking activity in coupled neighbors. Here, we describe a novel measurement for electrical synapse strength that directly evaluates the effect of synaptically transmitted activity on spike timing. This method, also applicable to neurotransmitter-based synapses, communicates the considerable strength of electrical synapses. For electrical synapses measured in rodent slices of the thalamic reticular nucleus and in simple model neurons, spike timing is modulated by tens of ms by activity in a coupled neighbor.

## Introduction

**Figure 1:**
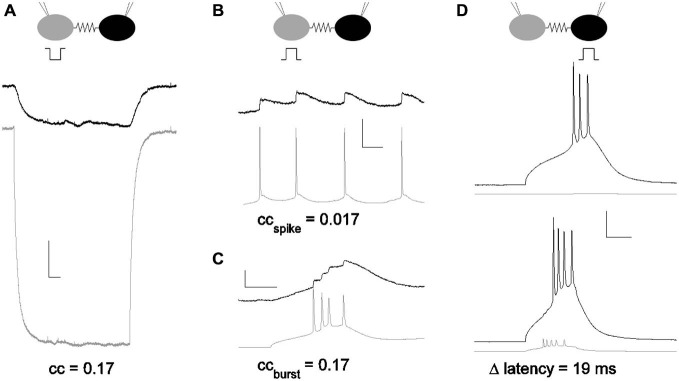
**Subthreshold and suprathreshold measurements for the strength of an electrical synapse. (A)** Coupling measured with current pulses. Coupling coefficients are a ratio of voltage deflections. Here, voltage deflections were initiated by a step in current delivered in one neuron (gray) that echoed in the coupled neuron (black). *cc* = 0.17 for the pair shown. Scale bar 1 mV, 50 ms. **(B)** Coupling coefficient measured by spike and spikelet amplitudes. Spikes were elicited in one neuron (gray) and spikelets in the coupled neighbor (black). Scale bar 2.5 mV (black,) 25 mV (gray), 25 ms. **(C)** Coupling coefficient measured by burst and burstlet amplitudes, for a longer burst event in one neuron (gray) and a burstlet in the coupled neighbor (black). Scale bar 2.5 mV (black), 25 mV (gray), 25 ms. **(D)** Coupling measured by latency changes. Modulation of spike latency δL was measured by comparing timing of spikes elicited in one cell alone (black; gray cell quiet), and with the coupled neighbor also driven to spike (gray). Scale bar 2 mV (gray), 20 mV (black), 25 ms. All data presented are from the same pair.

The strength of electrical synapses between gap junction-coupled neurons has traditionally been measured by the coupling coefficient (Bennett, 1966), which is the ratio of a steady, small voltage deflection transmitted from one cell to its neighbor across the synapse (Figure 1A). From the coupling coefficient, one can estimate the conductance of the synapse (Bennett, 1966; Fortier, 2010). Across the brain, average coupling coefficients measured from soma to soma vary from small (<0.05) in inferior olive (Devor and Yarom, 2002) and hippocampus (Zsiros and Maccaferri, 2005); to moderate, 0.1–0.15, in the thalamic reticular nucleus (Landisman et al., 2002) and cortex (Gibson et al., 1999); to even larger values, 0.2 in MesV (Curti et al., 2012). Coupling coefficients for physiological signals such as spikelets (Galarreta and Hestrin, 2001; Haas and Landisman, 2012) have been measured (Figures 1B,C), but are typically smaller than those measured for steady voltage deflections, due to their faster timecourses.

Coupling coefficients do not describe the role of electrical synapses in spiking, which varies with many factors, including excitability and intercellular distance. Yet that role is often substantial: in the dorsal cochlear nucleus, fusiform to stellate cell coupling is so effective as to control spiking in the stellates (Apostolides and Trussell, 2013), and coupling was shown to sharply increase the probability of spiking in coupled hypothalamic cells of the cichlid fish (Ma et al., 2014). In other populations, the impact of electrical synapses may be diminished by distance from a dendrodendritic synapse to the somatic integrator, while still driving a dendritic spike (Trenholm et al., 2014). Supra-threshold measures for the strength of electrical synapses have been used: correlation coefficients can be computed and compared for coupled pairs (Galarreta and Hestrin, 1999; Gibson et al., 1999; Long et al., 2002; Blatow et al., 2003; Haas and Landisman, 2012; Ma et al., 2014). However, correlation-based measures require both neurons to be activated by other inputs to similar states of firing; that firing must be steady or periodic; and correlation is measured and averaged over a period of time, encompassing several to many spikes, conditions which are more the exception than the rule *in vivo*. The question remains: how strong is an electrical synapse, in the context of spiking neurons? Specifically, what is the impact of an electrical synapse on the fundamental unit of neuronal communication—a spike?

In order to both quantify the strength of an electrical synapse and to provide a better basis for comparison to chemical synapses, we introduce a novel measure, δL, that expresses the efficacy of electrical synapses (Figure 1D). Herein, we explain the method and the simple test to measure it, compare it to traditionally used methods, and demonstrate its use in revealing the true strength of electrical synapses.

## Methods

The data used here have been previously reported (Sevetson and Haas, 2014). Horizontal slices 350–400 μm thick were obtained from Sprague-Dawley rats aged P11 – P14 of either sex. Rats were anesthetized using isofluorane and euthanized in accordance with federal and Lehigh IACUC animal welfare guidelines. Slices were cut and incubated in sucrose solution (in mM): 72 Sucrose, 83 NaCl, 2.5 KCl, 1 NaPO_4_, 3.3 MgSO_4_, 26.2 NaHCO_3_, 22 dextrose, 0.5 CaCl_2_. Slices were incubated at 36°C for 20 min and returned to room temperature until recording. The bath for solution for recording contained (in mM): 126 NaCl, 3 KCl, 1.25 NaH_2_PO_4_, 2 MgSO_4_, 26 NaHCO_3_, 10 dextrose and 2 CaCl_2_, 300–305 mOsm, saturated with 95% O_2_/5% CO_2_, The submersion recording chamber was held at 34°C (TC-324B, Warner Instruments). Micropipettes were filled with (in mM): 135 K-gluconate, 2 KCl, 4 NaCl, 10 HEPES, 0.2 EGTA, 4 ATP-Mg, 0.3 GTP-Tris, and 10 phosphocreatine-Tris (pH 7.25, 295 mOsm). For voltage-clamp measurements, 135 mM CsMSO_4_ was substituted for K-gluconate. Either 1 M CsOH or 1M KOH was used to adjust pH of the internal solution. The approximate bath flow rate was 2 ml/min. Voltages are reported as corrected for the liquid junction potential and bridge-balanced. The TRN was visualized under 5×, and pairs of TRN cells were identified at 40× IR-DIC optics (SliceScope, Scientifica). Signals were amplified and low-pass filtered at 8 kHz (MultiClamp, Axon Instruments), digitized at 20 kHz (lab-written Matlab routines controlling a National Instruments USB6221 DAQ board), and stored for offline analysis in Matlab (Mathworks, R2012a). Hodgkin-Huxley modeling was executed as previously described (Sevetson and Haas, 2014), simplified by setting calcium conductance to zero and using a single symmetrical electrical synapse. The three sodium conductances used were 60, 75 and 90 μS/cm^2^.

## Results

In response to depolarizing input of increasing amplitude, neurons spike with decreasing latency. This is a fundamental, common property of neuronal response. Recording from dual whole-cell patches of coupled neurons in thalamic reticular nucleus in acute brain slices, we performed an experiment designed to compare spike times in a neuron minimally stimulated from rest, with and without input from a neighbor across an electrical synapse, a paradigm that is repeatable in almost any pair of excitable cells.

**Figure 2:**
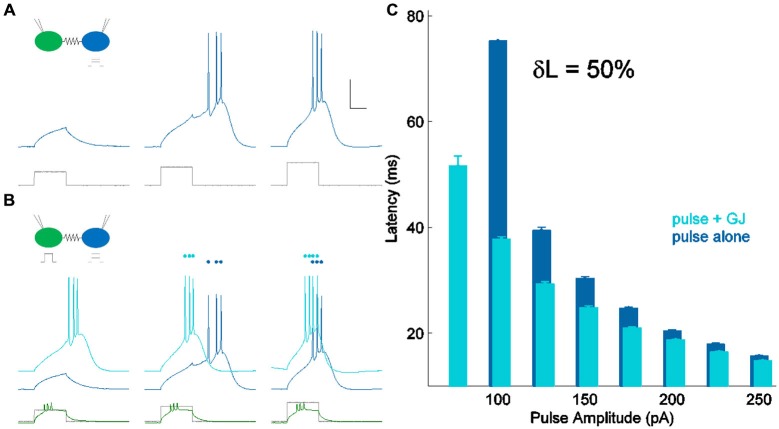
**Measuring δL, latency modulation**. **(A)** Spiking in one cell of a coupled pair (blue) in response to current pulses of increasing amplitude (lower, shown in gray). Scale bar 25 ms, 20 mV. The coupled cell was quiet and is not shown. **(B)** Spiking in the same cell (light blue) for the same current pulses as in **(A)** (lower, shown in gray), with the coupled neighbor also spiking (lower, shown in green). Responses from **(A)** are repeated, vertically offset for clarity (darker blue). **(C)** Latency of spiking in **(A)** (pulse alone) and **(B)** (pulse + GJ input) plotted against input amplitude. For peri-threshold inputs (100 pA) in this cell, δL, the percentage change in perithreshold spike latency, was 50%.

In all experiments, both cells were held near their resting voltage, at −70 mV. We used a set of 10 current steps with maximum amplitude of approximately 1 pA per MΩ of input resistance, delivered to one cell of a coupled pair through the recording electrode. Thus, for a cell of input resistance 250 MΩ, we delivered ten current pulses between 25 and 250 pA. We measured latency of spiking in that cell in two conditions: alone (Figure 2A), and with a suprathreshold input (~2× perithreshold) applied to the coupled neighbor (Figure 2B), driving it to spike before the first cell. Comparing the two sets of responses, we found that latency decreased when the synapse provided additional input (Figure 2C). We used this comparison to quantify the strength of the electrical synapse, for the smallest current step that consistently drove a spike in each cell. For the cell in Figure 2, the smallest input that reliably drove spiking was 100 pA; the cell did not spike for 75 pA of input without GJ input and δL was not calculable. Over 36 neurons, peri-threshold inputs were 93.5 ± 6.6 pA (mean ± SEM). This value was chosen for latency comparison in order to provide a realistic measurement of peri-threshold spike time modulation during synaptic input barrages *in vivo*.

Using these peri-threshold values of Δt_alone_ as the latency of the spike with a quiet neighbor and Δt_paired_ as the latency of the spike with an active neighbor, the quantity δL is expressed as a percentage change in latency:

$$\delta \text{L}=100*\frac{\Delta t_{\text{alone}}-\Delta t_{\text{paired}}}{\Delta t_{\text{alone}}}$$

**Figure 3:**
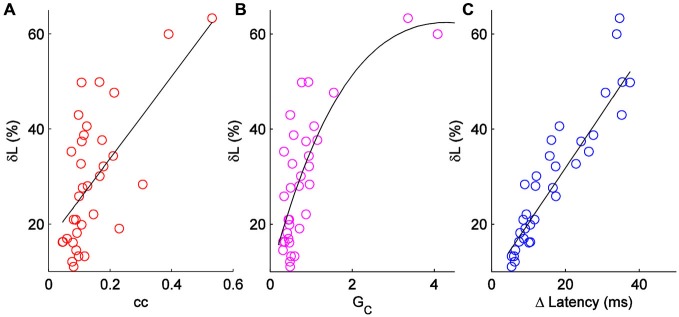
**Comparison of δL to other measures of electrical synapse strength. (A)** δL plotted against coupling coefficient cc in each direction for a set of *n* = 18 pairs. *R*^2^ = 0.38. **(B)** δL plotted against coupling conductance G_C_; *R*^2^ = 0.56. **(C)** δL plotted against absolute change in latency for each cell in 18 pairs.

δL has units of percentage in principle, δL can be negative. δL is unrelated to pulse input strength (*R^2^* = 0.07; not shown). δL is moderately correlated to coupling coefficients, and better related to the coupling conductances (Figures 3A,B) measured by hyperpolarizing current inputs. While input from the electrical synapse often converted an input that, alone, was subthreshold into a supra-threshold input (e.g., 75 pA in Figure 2C), δL does not include that effect. For our sample of electrical synapses, the average value of δL was 29.5 ± 2.2% (mean ± SEM, *n* = 36; Figure 3C). Applied to the average peri-threshold latency in our dataset of 56 ms, δL represents a difference in spike timing of 16.5 ms, a substantial difference on a neuronal timescale.

δL is experimentally as simple to measure as coupling coefficients, and provides an output that directly describes the impact of electrical synapses. This method can also be applied to chemical synaptic inputs. In order to compare electrical to chemical synapses, we performed the same experiment on a pair of somatosensory cortical (layer II) cells that were coupled through a glutamatergic synapse, with average response amplitude of 0.45 mV, an average strength for a chemical synapses (Feldmeyer et al., 2005). For this excitatory chemical synapse, the average decrease in peri-threshold latency was 1.4 ms, corresponding to a δL of 2.9%. This comparison demonstrates that in the context of spiking, electrical synapses are an order of magnitude more powerful than excitatory chemical connections.

We quantified δL before and after depressing the synapse by coordinated induced bursting activity (Haas et al., 2011) in a coupled pair for which initially, δL was 19.6%, representing a latency difference of 9.6 ± 0.3 ms at baseline. Depression of the synapse by 10.2% increased δL by 12.2%, to a final value of 22%, representing change in perithreshold latency of 12.0 ± 0.6 ms (*p* < 0.05). These values demonstrate that modest changes in electrical synaptic strength translate to physiologically meaningful changes in spike timing.

**Figure 4:**
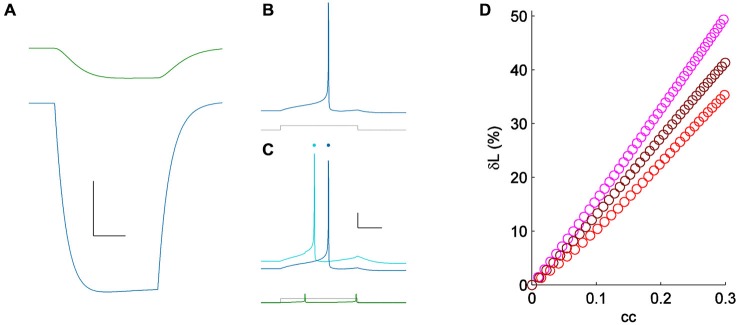
**(A)** Coupling demonstrated by a hyperpolarizing current pulse in a pair of simple Hodgkin-Huxley neurons; *cc* = 0.15. Scale bar 2 mV, 25 ms. **(B)** Spiking one of the model cells (blue) for a minimal input (lower, gray); the coupled neuron was quiet. **(C)** Spiking in the same cell (light blue) for the same current pulses as in **(A)** (lower, shown in gray), with the coupled neighbor also spiking (lower, shown in green). Responses from **(A)** are repeated, vertically offset, for clarity (dark blue). Scale bar 10 mV, 25 ms.** (D)** δL plotted against coupling coefficient in the modeled pair, for three values of excitability [sodium conductances of 60 (pink), 75 (maroon) and 90 (red) μS/cm^2^].

Because spiking in TRN neurons is heavily influenced by their low-threshold T current, we repeated measurement of δL in a coupled pair of simple Hodgkin-Huxley neurons (Figure 4). We used model neurons identical to those used in Sevetson and Haas (2014), but with zero *T* conductance, reducing the model to only sodium and potassium currents with a linear and symmetrical electrical synapse. For minimal stimuli, we applied step inputs that yielded initial latencies of ~75 ms (Figure 4B). For a moderate value of coupling (*cc* = 0.15), activity across the electrical synapse accelerated the model neuron’s spike time from 70–55 ms, or δL of 21% (Figure 4C). To test the dependence of neuronal excitability on δL, we varied sodium conductance in the model by 25–50% (Figure 4D). Using minimal stimuli in each set showed that while δL is weakly related to excitability, the strong modulatory effect of electrical synapses on spike times is reproduced by this simple model.

## Discussion

The method of quantifying electrical synapse strength that we introduce here, δL, reveals that electrical synapses are more powerful contributors to active spiking networks than subthreshold-based measurements of these synapses previously indicated. Together, this method and its application underline the strength of electrical synapses in shaping and altering spiking activity in coupled neurons across the brain.

By measuring the functional neuronal output, of spike times, δL captures interactions with or amplification of electrical synapses by postsynaptic nonlinear membrane conductances, such as the persistent sodium current (Curti et al., 2012; Haas and Landisman, 2012) or presynaptic effects, such as the afterhyperpolarizing currents relayed through gap junctions that delay spikes in coupled neighbors (Vervaeke et al., 2010). Like coupling coefficients, δL also can be used to quantify and compare asymmetry of electrical synapses (Sevetson and Haas, 2014).

In contrast to subthreshold-based measures, in principle δL can be measured by recording in a single neuron, while stimulating that neuron’s coupled neighbors through other (extracellular or optogenetic) means. Thus, δL offers a way to detect and quantify electrical synapses that is less technically demanding than performing paired recordings.

## Funding

JSH was supported in part as the Haddie Investigator, with a 2014 NARSAD Young Investigator Grant from the Brain and Behavior Research Foundation grant #21343, and by the Whitehall Foundation grant #2014-05-25.

## Conflict of Interest Statement

The author declares that the research was conducted in the absence of any commercial or financial relationships that could be construed as a potential conflict of interest.
